# A Service Evaluation: Survey of Staff Awareness of Physician Associate Role and Their Impact in Psychiatric Inpatient Wards in KMPT

**DOI:** 10.1192/bjo.2025.10486

**Published:** 2025-06-20

**Authors:** Shantala Satisha, Tenzin Lama, Naadiya Hall, Rachel Daly

**Affiliations:** Kent and Medway NHS Trust, Dartford, United Kingdom

## Abstract

**Aims:** This project aims to assess staff awareness of the Physician Associate (PA) role and the impact of PAs on an acute male psychiatric ward and a male psychiatric intensive care unit (PICU) in Kent and Medway NHS and Social Care Partnership Trust (KMPT).

PAs are integral to supporting the effective functioning of inpatient psychiatric wards and contributing to service development. At KMPT, there are currently five PAs working across 5 inpatient wards with 880 combined admissions last year. Bed pressures have increased annually, making consistent medical support essential. PAs, due to the nature of their role, can provide continuity and act as a key point of contact for staff.

Hypothesis: We hypothesize that staff may have limited awareness of the PA role due to its recent introduction in the trust, but that PAs will have a positive impact on patient care in mental health settings.

**Methods:** Data was collected through a survey to assess staff understanding of the PA role and its impact on patient care, collaboration, and team dynamics. Staff across various multidisciplinary team (MDT) roles, including the medical, nursing, occupational therapy, and psychology teams in the male PICU and a male acute ward were surveyed.

**Results:** A total of 32 responses were received.

Staff Awareness: 81.2% reported working closely with PAs daily. While 65.6% were very familiar with the PA role, 31.2% were somewhat familiar. Notably, 17.15% mistakenly believed PAs can prescribe medication and order ionizing radiation investigations.

Impact on Patient Care: 81.25% reported PAs made a significant impact in assessing and diagnosing physical health conditions, compared with 50% for mental health conditions. 64% said PAs significantly improved patient communication and engagement with carers.

Collaboration and Team Dynamics: 65.6% found PAs to be “very effective” in collaborating with the MDT. 58.3% agreed that PAs significantly reduced workload and administrative burden, improved continuity of care, and provided a consistent point of contact for ongoing care.

Overall Impact: 71.8% of staff reported a “very positive” impact of PAs; 28.1% felt it was “positive”; and 93.7% recommended expanding the role to other mental health services.

**Conclusion:** This survey shows most staff have daily contact with PAs; are familiar with their role; and believe PAs have a significant positive impact on patient care, collaboration, and continuity of care in inpatient mental health settings. However, there are knowledge gaps about specific limitations of the PA role, suggesting a need for further education to enhance staff understanding.

